# Effect of Dietary *n* − 3 Polyunsaturated Fatty Acids on Oxidant/Antioxidant Status in Macrosomic Offspring of Diabetic Rats

**DOI:** 10.1155/2014/368107

**Published:** 2014-06-02

**Authors:** B. Guermouche, N. A. Soulimane-Mokhtari, S. Bouanane, H. Merzouk, S. Merzouk, M. Narce

**Affiliations:** ^1^Laboratory of Physiology, Physiopathology and Biochemistry of Nutrition, Department of Biology, Faculty of Natural and Life Sciences, Earth and Universe, University of Abou Bekr Belkaid, BP 119, 13000 Tlemcen, Algeria; ^2^Department of Technical Sciences, Faculty of Engineering, University of Abou Bekr Belkaid, 13000 Tlemcen, Algeria; ^3^INSERM UMR 866, “Lipids Nutrition Cancer,” Faculty of Life, Earth and Environment Sciences, University of Burgundy, 21000 Dijon, France

## Abstract

The aim of this work was to determine the effect of dietary *n* − 3 PUFA on oxidant/antioxidant status, *in vitro* very low and low density lipoprotein (VLDL-LDL), and VLDL-LDL-fatty acid composition in macrosomic pups of diabetic mothers. We hypothesized that *n* − 3 PUFA would improve oxidative stress in macrosomia. Diabetes was induced in female Wistar rats fed with the ISIO diet (control) or with the EPAX diet (enriched in *n* − 3 PUFAs), by streptozotocin. The macrosomic pups were killed at birth (day 0) and at adulthood (day 90). Lipid parameters and VLDL-LDL-fatty acid composition were investigated. The oxidant/antioxidant status was determined by measuring plasma oxygen radical absorbance capacity (ORAC), hydroperoxides, carbonyl proteins, and VLDL-LDL oxidation. Macrosomic rats of ISIO fed diabetic mothers showed an increase in plasma and VLDL-LDL-triglycerides and VLDL-LDL-cholesterol levels and altered VLDL-LDL-fatty acid composition. Plasma ORAC was low with high hydroperoxide and carbonyl protein levels. The *in vitro* oxidizability of VLDL-LDL was enhanced in these macrosomic rats. The EPAX diet corrected lipid parameters and improved oxidant/antioxidant status but increased VLDL-LDL susceptibility to oxidation. Macrosomia is associated with lipid abnormalities and oxidative stress. *n* − 3 PUFA exerts favorable effects on lipid metabolism and on the oxidant/antioxidant status of macrosomic rats. However, there are no evident effects on VLDL-LDL oxidation.

## 1. Introduction

Maternal diabetes during pregnancy represents a significant risk of fetal overnutrition leading to fetal obesity or macrosomia, resulting from the combined effects of excessive transfer of maternal nutrients, and to fetal hyperinsulinemia as discussed by Mohammadbeigi et al. [1], which has been associated with the development of glucose intolerance, obesity, and diabetes during childhood and adulthood as discussed by Martin-Gronert and Ozanne [2]. However, the mechanisms by which fetal hyperinsulinemia and macrosomia predispose to adult diseases have not been well established.

We have previously used an animal model to explore the association between birth weight and the predisposition of macrosomic pups of diabetic dams to obesity development and the onset of adult diabetes as discussed by Merzouk et al. [3]. Macrosomic rats maintained accelerated postnatal growth and exhibited many of the metabolic characteristics that are typical of obese and diabetic subjects, including dyslipoproteinemia as discussed by Merzouk et al. [3].

On the other hand, oxidative stress has been implicated in several diseases such as atherosclerosis, diabetes, and obesity as discussed elsewhere [4, 5]. The increased oxidative damage may be a consequence of hyperglycemia and hyperlipidemia as discussed elsewhere [4, 6, 7].

The role of oxidative stress in the origin of type 1 diabetes or of insulin resistance in type 2 diabetes is also well established. Free radicals are involved in pancreatic beta-cell damage as discussed elsewhere [8, 9]. Hyperglycemia causes the dominance of oxidative stress over the antioxidative defense system, leading to oxidative DNA damage, which possibly contributes to pancreatic beta cells dysfunction, insulinoresistance, and the possible more pronounced hyperglycemia. This vicious cycle could possibly induce the deterioration into diabetes as discussed elsewhere [10, 11]. In addition, there is a considerable variation in the levels of lipid peroxides even in healthy subjects, and it has been proposed that people with elevated lipid peroxidation may be more prone to develop type 2 diabetes and cardiovascular disease as discussed by Halliwell [12].

It appears that not only hyperglycemia and hyperlipidemia may cause elevated peroxidation but also preexisting high rates of lipid peroxidation may predispose to diabetes.

The implication of oxidative stress in adult diseases and the predisposition of macrosomic offspring of diabetic mothers to these diseases raise the question of the possible link between oxidant/antioxidant status and the development of long-term metabolic abnormalities.

Using our animal model, we have previously reported altered oxidant/antioxidant status in adult macrosomic rats as discussed by Yessoufou et al. [13]. However, the oxidant/antioxidant status of these macrosomic rats was not investigated at birth; therefore the overall capacity of plasma samples to scavenge oxygen radicals (oxygen radical absorbance capacity, ORAC) assay as discussed by Cao et al. [14] will be better index of oxidant/antioxidant balance in these rats. Moreover, oxidizability of lipoproteins has been considered as contributory factor to oxidative stress in diabetes mellitus as discussed elsewhere [15, 16]. However, the relationships between LDL oxidizability, fatty acid composition, and oxidant/antioxidant status in macrosomia are still not clear.

Several reports have shown beneficial effects of *n* − 3 PUFAs in the reduction of plasma lipids and hyperglycemia, in addition to their anti-inflammatory, vasodilator, antihypertensive, and immunosuppressive effects as discussed by Lichtenstein et al. [16]. Fish oil diets have produced changes in lipoprotein composition in animal studies as discussed by Nassar et al. [17] and protect against lipid peroxidation in rats and humanswith diabetes as discussed elsewhere [18, 19].

We hypothesized that *n* − 3 PUFA could improve the oxidant/antioxidant status associated with macrosomia. To test our hypothesis, we used streptozotocin treatment of pregnant Wistar rats to induce mild maternal hyperglycemia, resulting in obese, hyperglycemic, and hyperinsulinemic offspring. We then produced a situation in which the fetal environment simulated that of a fetus in a poorly controlled human diabetic mother, which results in accelerated fetal growth in conjunction with fetal hyperinsulinemia. Pregnant rats and their macrosomic offspring were fed with an *n* − 3 PUFA-enriched diet. The time course of changes in lipid parameters and oxidant/antioxidant status was investigated by determining serum lipids, ORAC and variables for oxidative modified lipids (hydroperoxides) and proteins (carbonyl groups), the* in vitro* VLDL-LDL oxidizability, and its fatty acid composition. Glucose and insulin levels were also investigated in order to characterize the diabetic state of macrosomic offspring.

## 2. Methods and Materials

### 2.1. Animals and Experimental Protocol

Adult Wistar rats were obtained from (IFFA CREDO Lyon, France). After mating, the first day of gestation was estimated by the presence of spermatozoids in vaginal smears. Pregnant rats were housed individually in wood-chip-bedded plastic cages at a constant temperature (25°) and humidity (60 ± 5%) with a 12 h light/dark cycle. The rats had free access to water and were fed with two different diets, a control ISIO diet or an EPAX diet. The two diets composition was given in Table 1. EPAX-7010 was tightly sealed under a stream of nitrogen to avoid lipid oxidation and kept at 4°C.

The study was conducted in accordance with the national guidelines for the care and use of laboratory animals. All the experimental protocols were approved by the Regional Ethical Committee.

Diabetes was induced in 30 pregnant rats by intraperitoneal injection of streptozotocin (Sigma, St Louis, MO, USA) (40 mg/kg of body weight) in 0.1 mol/L citrate buffer (pH 4.5) on day 5 of gestation. Twenty pregnant rats were injected with citrate buffer alone as a control group. On days 12, 16, 18, and 20 of gestation, maternal blood was collected for glucose concentration by cutting off the tip of the tail and squeezing it gently. Pregnant rats with plasma glucose levels between 5.55 and 16.65 mmol/L were assigned as mildly hyperglycemic as cited elsewhere [3, 20, 21] and were included in the present study. At delivery, pups from the streptozotocin-treated dams whose birth weights were 1.7 S.D. (above the 90th percentile) greater than the mean birth weight of the control pups were classified as macrosomic pups as cited elsewhere [3, 20–22] and included in the present study. The mean birth weight of the control pups was 5.28 ± 0.50 g. Therefore experimental pups with birth weight >6.13 g were included as macrosomic in the present study. The success rate of obtaining macrosomic pups was 62% in the group fed with the ISIO diet and 55% in the group fed with the EPAX diet. The mean birth weight of the macrosomic pups was 7.86 ± 0.44 g. These macrosomic pups were hyperglycemic and hyperinsulinemic at birth. The nonmacrosomic offspring of diabetic mothers were excluded, since maternal diabetes related to fetal macrosomia was the criterion for our experimental population selection. However, these normal-sized offspring of diabetic mothers were not hyperinsulinemic at birth, had normal growth rates, and showed no significant differences from controls for serum lipids. Twenty newborn rats from each group (control and experimental) and each diet (ISIO and EPAX) were killed by decapitation, and blood was collected. The remaining macrosomic and control pups from each diet were left with their mothers. Litter sizes were kept between six and eight pups per nursing dam to maintain a similar postnatal intake during the suckling period. Pups were weighed weekly up to 12 weeks of age. Pups were weaned at 3 weeks of age, housed three rats per cage, and allowed the control or EPAX diet and water* ad libitum*.

### 2.2. Blood Samples

At birth, rats were killed by decapitation and blood was collected. At day 90, six pups from each group were anaesthetized with intraperitoneal injection of sodium pentobarbital (60 mg/kg of body weight). The abdominal cavity was opened and the whole blood was drawn from the abdominal aorta into heparinized tubes. Plasma was obtained by low-speed centrifugation (1000 ×g for 15 min) and was used immediately for lipid, lipoprotein, and oxidative stress parameters determinations.

#### 2.2.1. Very Low and Low Density Lipoprotein Isolation

Several authors have used combined VLDL-LDL fraction to test lipoprotein oxidation in rats as cited by Frenoux et al. [23]. The VLDL-LDL fraction was obtained by precipitation with dextran sulfate (0.91 g/L) and MgCl_2_ (91 mmol/L) as described by SJOBLOM and EKLUND as cited by Sjoblom and Eklund [24].

#### 2.2.2. Lipid Determination

VLDL-LDL-triglyceride and total cholesterol contents were measured by means of Boehringer kits (Mannheim, Germany), using enzymatic methods.

#### 2.2.3. Determination of Plasma Vitamin E Levels

Plasma *α*-tocopherol (vitamin E) was determined by reverse phase HPLC according to the method of Zaman et al. [25]. The HPLC system comprised a Dionex separation module (P680 HPLC Pump and ASI-100 Automated Sample Injector, Dionex, Aix-en-Provence, France) and a Jasco fluorometric detector (Jasco, Nantes, France). This method was used to quantify vitamin E level in a single chromatographic run with an internal standard, Tocol (Lara Spiral, Couternon, France), added for estimation of recovery. The stationary phase was constituted by grafted silica (C18 column, HP ODS Hypersil C18; 200 mm × 4,6 mm; Lara Spiral, maintenance temperature of analytical column, 35°C). The mobile phase was a mixture of methanol/water (98/2, v/v) at a flow rate of 1 mL/min. Vitamin E was extracted by hexane, dried under nitrogen, and resuspended in methanol. The HPLC peaks were detected by a UV detector at 292 nm. Representative chromatograms were obtained by injecting standard solution. In order to evaluate the daily performance of HPLC system, the external standard was injected every day in the beginning, middle, and at the end of the chromatographic system. The external standard was injected every day for daily system suitability testing to ensure data validity. Neither internal adjustment nor response curve for quantitation is intended here. Then, representative chromatograms were obtained by injecting standard solutions.

#### 2.2.4. Scavenging Capacity of Plasma

Plasma ORAC was measured according to Semenkovich and Heinecke [15]. A fluorescent protein, allophycocyanin (APC), was used in this assay as cited by Courderot-Masuyer et al. [26]. ORAC employs the oxidative loss of the intrinsic fluorescence of APC. APC fluorescence decay shows a lag or retardation in the presence of antioxidants, related to the antioxidant capacity of the sample. The reaction mixture (2 mL) contained a final concentration of 37.5 nmol/L APC in 75 mmol/L phosphate buffer (pH 7.0) at 37°C in the absence (blank) or presence of 20 *μ*L of Trolox (1 *μ*mol/L) or plasma, respectively. The reaction was initiated by the introduction of 9 *μ*mol/L of CuSO_4_ and 0.3% H_2_O_2_ as redox catalysts. This reaction was followed spectrophotometrically by the decrease in fluorescence at 651 nm emission and 598 nm excitation, using a spectrofluorometer SFM25 Kontron. Trolox was used as a reference antioxidant for calculating the ORAC values, with one ORAC unit defined as the net protection area provided by 1 *μ*mol/L final concentration of Trolox. ORAC value of the samples was calculated as ORAC ((*A* sample −* A* blank)/(*A* Trolox −* A* blank));* A* refers to the area under the quenching curve of APC.

#### 2.2.5. Determination of Hydroperoxide Levels

Plasma hydroperoxides were measured by the ferrous ion oxidation-xylenol orange assay (Fox2) in conjunction with a specific ROOH reductant, triphenylphosphine (TPP), according to the method of Nourooz-Zadeh et al. [27]. Fox2-reagent was obtained as the commercially available material from Pierce (Peroxoquant methanol-compatible formulation kit, Rockford, IL, USA). This method is based on the principle of the rapid peroxide-mediated oxidation of Fe^2+^ to Fe^3+^ under acidic conditions. The latter, in the presence of xylenol orange, forms a Fe^3+^-xylenol orange complex which can be measured spectrophotometrically at 560 nm. Hydroperoxide content in the samples was determined as a function of the mean absorbance difference of samples with and without elimination of ROOH by TPP. Calibration was done with standard peroxides such as hydrogen peroxide.

#### 2.2.6. Determination of Plasma Carbonyl Proteins

Plasma carbonyl proteins (marker of protein oxidation) were assayed by 2,4-dinitrophenylhydrazine reaction according to Levine et al. [28]. 50 *μ*L of plasma was incubated for 1 hour at room temperature with 1 mL of 2 g/L dinitrophenylhydrazine (DNPH) in 2 mol/L HCl or 2 mol/L HCl as control blank. Next, proteins were precipitated with 200 *μ*L of 500 g/L trichloroacetic acid (TCA) and washed three times with 1 : 1 (v/v) ethanol: ethyl acetate and three times with 100 g/L TCA. The final precipitate was solved in 6 mol/L guanidine and the spectrum DNPH versus HCl controls was followed at 350–375 nm. The concentration of carbonyl groups was then calculated using 21,5 (mmol/L) cm^−1^ as the extinction coefficient for aliphatic hydrazones.

#### 2.2.7. VLDL-LDL Susceptibility to* In Vitro* Oxidative Stress

VLDL-LDL fractions were diluted to a final concentration of 50 *μ*g/mL protein using PBS and incubated at 37°C for 18 h with a freshly prepared CuSO_4_ solution added to a final concentration of 10 *μ*mol/L as reported by Rabini et al. [29] and Nourooz-Zadeh et al. [27]. The degree of lipoprotein oxidation was determined by the measurement of hydroperoxide levels before and after the peroxidative stress. VLDL-LDL susceptibility to oxidation is measured as differential hydroperoxide content before and after incubation with CuSO_4_.

#### 2.2.8. VLDL-LDL Protein Modification

The percentage reduction in free lysine groups present in VLDL-LDL after exposure to oxygen radicals was measured using trinitrobenzenesulfonic acid, as previously described and according to Habeeb [30]. Briefly, native and oxidized VLDL-LDL (50 *μ*g of protein) were added to 1 mL of NaHCO_3_ (4% solution at pH 8.4) and 50 *μ*L of trinitrobenzenesulfonic acid (0.1% solution) and heated for 70 min at 37°C. After this step, the absorbance at 340 nm was measured and compared with results obtained for native VLDL-LDL.

#### 2.2.9. VLDL-LDL-Fatty Acid Composition

VLDL-LDL-fatty acid content was determined by extracting lipids according to the method of Folch et al. [31]. For the determination of the fatty acid content in LDL, lipids were extracted by the method of Folch et al. [31]. After saponification with NaOH/methanol 0.5 N, fatty acids were transmethylated by boron trifluoride/methanol (14%) at 80°C for 15 min. Fatty acid methyl esters were analyzed by gas liquid chromatography as cited by Habeeb [30] using a Becker gas chromatograph (Becker Instruments, Downers Grove, IL, USA) equipped with a 50 m capillary glass column packed with Carbowax 20 m (Spiral-RD, Couternon, France). C_17:0_ methyl ester was used as an internal standard. Identification of different fatty acids was performed by comparison of relative retention times with those of commercial standards. Areas were calculated with an ENICA 21 Integrator (Delsi Instrument, Suresnes, France).

### 2.3. Statistical Analysis

Results are expressed as means ± SD. Significant differences among the groups were analyzedstatistically by ANOVA. Significant differences between macrosomic and control rats and between ISIO and EPAX diets at each age were assessed using Student's *t* test. In order to reduce the animals number used in this study, power analysis was used and set at 80%. These calculations were performed using Statistica version 4.1 (STATSOFT). Differences were considered statistically significant at *P* < 0.05.

## 3. Results

### 3.1. VLDL-LDL-Lipids Contents (Table 2)

The macrosomic pups of mothers fed with the ISIO diet, at birth and day 90, had an increase in the levels of VLDL-LDL-TG and TC compared with control rats. The EPAX diet significantly decreased these parameters in macrosomic rats to levels observed in controls.

### 3.2. Fatty Acid Composition of VLDL-LDL-Lipids (Table 3)

The macrosomic pups from diabetic mothers fed with the ISIO diet showed a significant increase in VLDL-LDL-saturated fatty acids (SFA) and C_20:4*n*−6_ and a decrease in VLDL-LDL-C_18:2*n*−6_ and C_22:6*n*−3_ levels compared with control pups. However, macrosomic rats from diabetic mothers fed with the EPAX diet showed no difference compared with the control rats at day 0 and day 90.

At birth, macrosomic rats on the EPAX diet compared with rats on the ISIO diet had a significant increase in VLDL-LDL-C_18:2*n*−6_, C_20:5*n*−3_, and C_22:6*n*−3_, which was associated with a decrease in SFA and C_20:4*n*−6_ levels. At day 90, we noted only an increase in VLDL-LDL-C_18:2*n*−6_, C_20:5*n*−3_, and C_22:6*n*−3_.

### 3.3. Plasma Oxidative Stress Parameters (Figure 1)

The levels of vitamin E were not affected in macrosomic rats at birth or at day 90. Similarly, the EPAX diet did not influence vitamin E levels.

Plasma total antioxidant status (ORAC) was lower in macrosomic rats compared to controls at days 0 and 90, regardless of type of diet. However the EPAX diet enhanced scavenging capacity of plasma in macrosomic rats at birth and at adulthood.

Plasma hydroperoxide levels (indicator of lipid peroxidation) were increased in macrosomic rats compared to controls at day 0 and day 90, in both diets. A significant reduction in hydroperoxides was noted in macrosomic EPAX-fed rats compared to ISIO fed.

Similarly, plasma carbonyl protein contents were higher in macrosomic versus control rats. The EPAX diet reduced the marked increases in carbonyl proteins induced by macrosomia.

### 3.4. *In Vitro* VLDL-LDL Susceptibility to Oxidation (Table 4)

The basal hydroperoxide levels were significantly increased in macrosomic rats and reduced by the EPAX diet, reflecting plasma changes. Significantly increased hydroperoxide levels were found in Cu^2+^-catalyzed oxidized LDL with respect to nonoxidized VLDL-LDL in both macrosomic and control rats. The* in vitro* oxidative stress induced by Cu^2+^ incubation resulted in a significantly greater increase in hydroperoxide levels in VLDL-LDL from macrosomic rats than in VLDL-LDL from controls, regardless of the type of diet. However, the extent of hydroperoxide formation was enhanced by the EPAX diet in macrosomic rats at birth and at day 90. The percentage of lysine group reductions, after* in vitro* CuSO_4_ induced LDL oxidation, was enhanced in macrosomic rats, regardless of the age or the diet fed. Feeding the EPAX diet did not influence the reduction of lysine groups in proteins of oxidized VLDL-LDL.

## 4. Discussion

Considerable interest has been generated over the last decade in the potential role of factors that affect fetal growth, thus resulting in the in-depth studies of chronic diseases including obesity, diabetes mellitus, and atherosclerosis. Previous studies have shown that fetal overnutrition or macrosomia is associated with the acquisition of later risk factors for chronic diseases as discussed by Martin-Gronert and Ozanne [2]. Disturbances in the antioxidant defense system in obesity, diabetes mellitus, and atherosclerosis have been reported as discussed elsewhere [4, 5]. To investigate the long-term effects of macrosomia, especially with regard to the oxidant/antioxidant status, we used streptozotocin treatment of pregnant Wistar rats to induce mild hyperglycemia resulting in obese, hyperglycemic, and hyperinsulinemic offspring. Because *n* − 3 PUFA exerts beneficial effects against oxidative stress as cited elsewhere [18, 19, 31], it was thought worthwhile to study the effect of an *n* − 3 PUFA-rich diet on the oxidant/antioxidant status of macrosomic rats.

We have previously shown that the macrosomic offspring were hyperglycemic and hyperinsulinemic at birth and had accelerated growth compared to offspring of control rats, regardless of maternal diet as cited by Martin-Gronert and Ozanne [2]. Indeed, the macrosomic pups of ISIO fed mothers had hyperlipidemia reflected by increased VLDL-LDL-cholesterol and triglyceride levels which is in agreement with previous studies as discussed elsewhere [3, 22, 32].

Maternal hyperglycemia enhanced NEFA transfer from the diabetic mother and fetal hyperinsulinemia have all been suggested to contribute to fetal hyperlipidemia as discussed elsewhere [1, 33, 34]. Pronounced changes in fatty acid composition in VLDL-LDL-lipids were also observed at birth in these macrosomic rats. The newborns of diabetic rats fed with the ISIO diet had a significant increase in VLDL-LDL-SFA and C_20:4*n*−6_ and a decrease in VLDL-LDL-C_18:2*n*−6_ and VLDL-LDL-C_22:6*n*−3_ levels compared with control newborns. Increased fatty acid synthesis or reduced fatty acid oxidation resulting from fetal hyperinsulinemia and high Δ6-desaturase activity might explain these modifications, as we have previously suggested as discussed elsewhere [32, 35].

These observations corroborated our previous studies as cited elsewhere [13, 32]. At day 90, the major findings concerning VLDL-LDL-lipid fatty acid composition were a significant decrease in C_18:2*n*−6_ and C_20:4*n*−6_ in ISIO fed macrosomic rats compared to their controls. These findings are in agreement with our previous studies according to Slover and Lanza [32].

Feeding the EPAX diet to macrosomic rats during adulthood induced a significant decrease in VLDL-LDL-cholesterol and VLDL-LDL-triglyceride levels, in agreement with our previous results as cited elsewhere [13, 32].

Thus, the EPAX diet attenuated hyperlipidemia, a long-term complication of macrosomia, in offspring of diabetic dams. This observation is in accordance with previous findings, which have shown that *n* − 3 PUFA-enriched diets decrease both plasma triglyceride and cholesterol levels according to Pelikánová et al. [36]. Indeed, the dietary EPAX intake induced a large increase in EPA and DHA followed by a decrease in C_18:2*n*−6_ or C_20:4*n*−6_ in VLDL-LDL-lipids, confirming previous data cited by Cao et al. [14]. As far as oxidant/antioxidant status is concerned, our results provide evidence that oxidative stress is involved in macrosomia.

Oxidative stress occurs when the generation of free radicals (i.e., substances with one or more impaired electrons) exceeds the capacity of antioxidant defense mechanisms. Free radical-induced damage of cells, lipids, and proteins has been linked to the etiology of a number of disease states as discussed elsewhere [4, 5]. As free radicals may result in antioxidant depletion, one logical approach to assessment of oxidative stress lies in evaluation of the total antioxidant capacity (ORAC). Our data revealed that ORAC was decreased in macrosomic rats either ISIO or EPAX fed compared to their respective controls, at birth and at day 90. These results are in agreement with our previous results cited by Yessoufou et al. [13].

An increase in free radical production and antioxidant depletion was recently described in macrosomic rats by Yessoufou et al. [13], and in type 1 or type 2 diabetes in humans by Bensellam et al. [37]. In fact, there are complex antioxidant defense systems, including both enzymatic and nonenzymatic components, against the effects of oxygen free radicals on biological macromolecules such as proteins and lipids.

Despite normal levels of vitamin E, there is a lipophilic antioxidant interfering with the chain reaction of lipid peroxidations according to Sirtori and Galli [38] and a reduction of ORAC associated with the increase of hydroperoxide levels in macrosomic rats at birth and at day 90. Hydroperoxides were measured as a marker of lipid peroxidation. A number of studies have assessed elevated levels of hydroperoxides in plasma from both type 1 and type 2 diabetic patients, resulting from their hyperglycemia as cited by Merzouk et al. [5]. It is well known that increased glucose availability enhances free radical production and depresses the natural antioxidant defenses as discussed elsewhere [4, 11]. In addition, protein carbonylation was increased in macrosomic rats compared to their controls, at birth and day 90.

Protein carbonyl groups are introduced via oxidation of proteins and can be used as markers for oxidatively modified proteins according to Sirtori and Galli [38]. Protein carbonyl contents were found to be increased in clinical and experimental diabetes as discussed elsewhere [4, 10, 39]. Oxidatively modified proteins have been suggested to be a sign of tissue damage caused by oxidative stress, carbohydrate overload, or both as cited elsewhere [38, 40]. The protein carbonyl's contents reflect the amount of oxidative stress the animal has been exposed to during a long time period according to Medina et al. [39].

Taken together, our results suggested that oxidative stress occurred during intrauterine life, persisted through adulthood in macrosomic rats, and might be related to hyperglycemia.

Our data indicated that the EPAX diet enhanced ORAC values and reduced hydroperoxide and carbonyl protein contents in macrosomic rats. Thus, a PUFA diet improved oxidant/antioxidant status and reduced the oxidative stress induced by macrosomia, which is indicative of the beneficial effect of EPAX according to Yessoufou et al. [13].

A major finding in this study was that VLDL-LDL isolated from macrosomic rats was more oxidized and became more extensively oxidized under the experimental condition utilized in this study. Although the factors determining the susceptibility of VLDL-LDL to oxidation are not thoroughly understood, CuSO_4_ oxidation of lipoproteins has been widely used to examine VLDL-LDL oxidation as discussed elsewhere [5, 38, 40, 41]. Oxidative modification of VLDL-LDL induced by CuSO_4_ *in vitro* was assessed by monitoring the formation of hydroperoxides and the reduction of free lysine groups (trinitrobenzene sulfonic acid assay). The difference in both susceptibility to oxidation and extent to oxidation, between VLDL and LDL from macrosomic and control rats, was substantial and reflected greater oxidative modification of both lipid and protein components of VLDL-LDL. The extent of hydroperoxide formation was greater in VLDL-LDL from macrosomic rats, at birth and at day 90, regardless of type of diet. These data are consistent with several other studies examining VLDL-LDL oxidation in diabetes according to Merzouk et al. [5].

In our study, VLDL-LDL was rapidly isolated and utilized immediately in oxidation experiments, thereby reducing autooxidation of samples during their preparation and storage. In addition, samples from macrosomic and control rats were isolated and run together to avoid a systematic bias resulting from interassay variation over time. Since macrosomic rats were hyperglycemic at birth and at day 90, the clear demonstration of enhanced susceptibility to oxidation of VLDL-LDL might be related to hyperglycemia, as occurred in diabetes as described elsewhere [4, 5, 10, 42]. Lipid alterations in ISIO-fed macrosomic rats, such as high TG, VLDL-LDL-TC, and VLDL-LDL-TG levels, could influence the susceptibility of VLDL-LDL oxidation. These notions are well documented in diabetes as discussed elsewhere [5, 41, 43].

Hyperglycemia leads to modifications of proteins by nonenzymatic glycation as described elsewhere [4, 44], forming advanced glycation end products (AGE), which are sources of free radical production according to Liguori et al. [44]. One product of both oxidation and glycation of proteins is carboxymethyl lysine, a structurally defined AGE, quantitatively most formed* in vitro* as cited by Feillet et al. [45]. A significantly greater decrease in free lysine groups was seen in VLDL-LDL from macrosomic rats than from controls, at birth and at day 90. The percentage of free lysine groups after* in vitro* VLDL-LDL oxidation is considered as an indicator for carboxymethyl lysine formation which is a biomarker for the glycation and oxidative damage to proteins in diabetes according to Feillet et al. [45].

Another important finding in this study was that the EPAX diet had no protective effect on VLDL-LDL oxidation. In fact, the EPAX diet increased the susceptibility of VLDL-LDL to* in vitro* copper-induced oxidation. The susceptibility of VLDL-LDL to oxidation depends mainly on their PUFA contents and their concentrations of antioxidants as described elsewhere [46, 47].

Because vitamin E levels were not different between macrosomic and control rats and were not affected by the EPAX diet, we assumed that the increased susceptibility of VLDL-LDL to oxidation in macrosomic rats fed with this diet was due to their enhanced DHA and EPA percentages. VLDL-LDL susceptibility to oxidation could also be a function of* in vivo* glycosylation since EPAX-fed macrosomic rats were still hyperglycemic. The susceptibility of VLDL-LDL to oxidation* ex vivo* has been used by many groups to examine possible mechanisms whereby VLDL-LDL might contribute to oxidative stress and pathology* in vivo* as cited by Esterbauer et al. [48]. The relevance of VLDL-LDL oxidizability* ex vivo* to oxidative stress* in vivo*, however, is still not clear. Some studies have shown a relationship between lipoprotein oxidizability* ex vivo* and disease risk, but others have not, and there is a sizeable body of literature that is not compatible with an important role for* in vitro* lipoprotein oxidizability in disease risk according to Aldini et al. [49]. We confirm that this test is not suitable for testing the beneficial effects of *n* − 3 PUFA on oxidative stress in macrosomia. The study of VLDL-LDL in isolation* in vitro* may not fully represent the situation* in vivo*.

In conclusion, macrosomia is associated with lipid abnormalities and oxidative stress at birth and throughout adulthood. *n* − 3 PUFAs exert favorable effects on lipid metabolism and on the oxidant/antioxidant status of macrosomic rats. However, their effects on* in vitro* VLDL-LDL oxidation were not evident and did not represent the* in vivo* condition. *n* − 3 PUFA supplementation could lead to the improvement of the oxidant/antioxidant status in macrosomia of diabetic pregnancy.

## Figures and Tables

**Figure 1:**
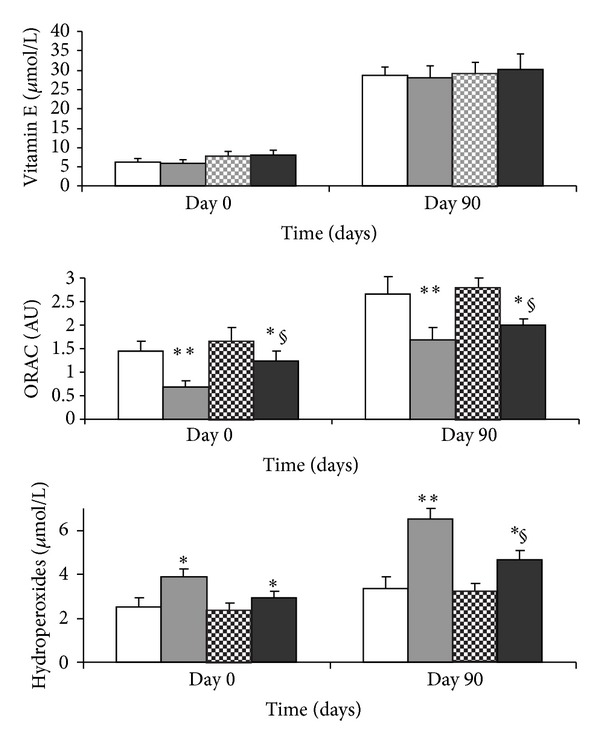
Plasma oxidative stress markers in control and macrosomic rats. Values are means ± SD, *n* = 8 rats. Day 0: birth; day 90: adulthood; EPAX: EPAX diet; ISIO: ISIO diet; ORAC: oxygen radical absorbance capacity. Significant differences between macrosomic and control rats at each age are indicated as *P < 0.05; **P < 0.01. Significant different values between control diet and EPAX diet groups at each age are indicated as ^§^ *P* < 0.05; ^§§^ *P* < 0.01.

**Table 1:** Composition of experimental diets.

	ISIO	EPAX
Components (g/100 g)		
Casein	20	20
Starch	58.7	58.7
Saccharose	5	5
Cellulose	5	5
Minerals	4	4
Vitamins	2	2
Methionine	0.3	0.3
Fat (ISIO)	5	0
Fat (EPAX)	2.5	2.5
Energy content (Kcal/100 g)	379.8	379.8
Fatty acid composition (mg/g)		
SFA	9.4	4.26
MUFA	18.50	9.07
*n* − 6 PUFA	21.30	12.6
*n* − 3 PUFA	0.83	24.59
EPA	—	22.2
DHA	—	2

DHA: docosahexaenoic acid; EPA: eicosapentaenoic acid; MUFA: monounsaturated fatty acids; SFA: saturated fatty acids. Fatty acid composition was analyzed by gas liquid chromatography as mentioned in Methods and Materials Section. Minor polyunsaturated fatty acids were not presented (<0.5).

**Table 2:** VLDL-LDL-lipids in control and macrosomic rats.

	Day 0	Day 0	Day 90	Day 90
	Control	Macrosomic	Control	Macrosomic
VLDL-LDL TG (mmol/L)				
ISIO	0.08 ± 0.03	0.24 ± 0.02*	0.20 ± 0.05	0.44 ± 0.04*
EPAX	0.13 ± 0.03	0.13 ± 0.03^§^	0.22 ± 0.03	0.25 ± 0.04^§^
VLDL-LDL TC (mmol/L)				
ISIO	1.27 ± 0.31	2.19 ± 0.19*	2.14 ± 0.12	2.98 ± 0.12*
EPAX	1.40 ± 0.30	1.74 ± 0.20^§^	2.27 ± 0.12	2.20 ± 0.18^§^

Values are mean ± SD; *n* = 8 rats. Day 0: birth; day 90: adulthood; EPAX: EPAX diet; ISIO: ISIO diet; LDL: low density lipoprotein; TC: total cholesterol; TG: triglyceride; VLDL: very low density lipoprotein. Data were analysed statistically by ANOVA. Significant differences between macrosomic and control rats and between ISIO and EPAX diets at each age were assessed using Student's *t* test. *P < 0.05 compared with the control group. ^§^ *P* < 0.05 compared with the group fed with the ISIO diet.

**Table 3:** VLDL-LDL-fatty acid compositions from control and obese offspring.

	Day 0	Day 0	Day 90	Day 90
	Control	Macrosomic	Control	Macrosomic
SFA				
ISIO	35.08 ± 1.54	42.11 ± 2.64**	37.20 ± 2.48	39.97 ± 2.91
EPAX	35.3 ± 2.97	35.05 ± 1.11^§^	34.95 ± 3.59	37 ± 2.34
MUFA				
ISIO	24.95 ± 2.14	21.67 ± 2.25	19.61 ± 1.86	18.52 ± 2.75
EPAX	23.06 ± 1.97	22.56 ± 2.39	20.31 ± 2.34	19.31 ± 1.76
18:2 (*n* − 6)				
ISIO	13.25 ± 2.65	6.18 ± 0.86**	18.72 ± 2.01	6.53 ± 1.63**
EPAX	10.07 ± 1.24	9.6 ± 0.78	15.82 ± 1.83	10.19 ± 1.73^§§^
20:4 (*n* − 6)				
ISIO	21.25 ± 1.09	26.63 ± 1.17**	20.17 ± 1.97	10.75 ± 0.89**
EPAX	18.26 ± 1.01^§^	17.85 ± 1.24^§§^	12.84 ± 1.10^§§^	11.34 ± 0.78
EPA 20:5 (*n* − 3)				
ISIO	1.07 ± 0.48	1.51 ± 0.24	1.18 ± 0.24	1.26 ± 0.33
EPAX	5.63 ± 0.62^§§^	5.66 ± 0.85^§§^	8.05 ± 1.01^§§^	7.84 ± 1.01^§§^
DHA 22:6 (*n* − 3)				
ISIO	1.26 ± 0.74	0.58 ± 0.24*	1.97 ± 0.26	1.62 ± 0.27
EPAX	6.09 ± 0.82^§§^	7.35 ± 0.38^§§^	5.85 ± 0.62^§§^	7.27 ± 0.83^§§^

Values are expressed as a percentage of the total fatty acids and are mean ± SD; *n* = 8 rats. Day 0: birth; day 90: adulthood; DHA: docosahexaenoic acid; EPA: eicosapentaenoic acid; EPAX: EPAX diet; ISIO: ISIO diet; MUFA: monounsaturated fatty acids; SFA: saturated fatty acids. Data were analysed statistically by ANOVA. Significant differences between macrosomic and control rats and between Isio-4 and EPAX diets at each age were assessed using Student's *t* test. *P < 0.05 and  **P < 0.01 compared with the control group. ^§^ *P* < 0.05 and ^§§^ *P* < 0.01 compared with the group fed with the ISIO diet.

**Table 4:** *In vitro* oxidation of VLDL-LDL isolated from control and macrosomic rats.

	Day 0	Day 0	Day 90	Day 90
	Control	Macrosomic	Control	Macrosomic
Basal oxidation hydroperoxide (nmol/mg VLDL-LDL protein)				
ISIO	15.29 ± 1.38	26.77 ± 1.05**	27.50 ± 1.22	57.95 ± 2.17**
EPAX	13.74 ± 1.02	19.94 ± 1.11^∗§^	25.95 ± 1.15	43.18 ± 2.08^∗§§^
Oxidation with CuSO_4_ Hydroperoxide (nmol/mg VLDL-LDL protein)				
ISIO	46.51 ± 3.42	83.87 ± 4.36**	79.94 ± 5.54	162.52 ± 8.48**
EPAX	50.36 ± 2.33	102.96 ± 5.25^∗§^	90.27 ± 6.38	296.98 ± 9.41^∗∗§§^
Oxidation with CuSO_4_ TNBS (free lysine groups, %)				
ISIO	−17.25 ± 1.04	−29.5 ± 1.26**	−30.8 ± 2.40	−42.67 ± 2.35**
EPAX	−15.16 ± 1.35	−27.6 ± 2.18**	−31.35 ± 2.32	−45.98 ± 2.29**

Values are mean ± SD; *n* = 8 rats. Day 0: birth; day 90: adulthood; EPAX: EPAX diet; LDL: low density lipoprotein; ISIO: ISIO diet; TNBS: trinitrobenzene sulfonic acid assay; VLDL: very low density lipoprotein. Data were analysed statistically by ANOVA. Significant differences between macrosomic and control rats and between ISIO and EPAX diets at each age were assessed using Student's *t* test. *P < 0.05 and **P < 0.01 compared with the control group. ^§^ *P* < 0.05 and  ^§§^ *P* < 0.01 compared with the group fed with the ISIO diet.

## Abbreviations

DHA: Docosahexaenoic acid
EPA: Eicosapentaenoic acid
LDL: Low density lipoprotein
ORAC: Oxygen radical absorbance capacity
PUFA: Polyunsaturated fatty acids
TC: Total cholesterol
TG: Triglyceride
VLDL: Very low density lipoprotein.
